# Research on the development and testing methods of physical education and agility training equipment in universities

**DOI:** 10.3389/fpsyg.2023.1155490

**Published:** 2023-06-28

**Authors:** Baohua Tan, Shihao Tian, Enpu Wang, Lu Xiao, Kan Cao, Beitian Zheng, Lina Luo

**Affiliations:** ^1^School of Science (School of Chip Industry), Hubei University of Technology, Wuhan, China; ^2^National “111 Research Center” Microelectronics and Integrated Circuits, Hubei University of Technology, Wuhan, China; ^3^School of Physical Education, Hubei University of Technology, Wuhan, China

**Keywords:** sensitive qualities, agile training, mesh network, reactive time, sports equipment

## Abstract

**Introduction:**

Because of the problems of insufficient funds and traditional training methods in college sports agile training, an agile training system based on a wireless *ad hoc* network was developed to evaluate the effect of improving the sensitive quality of ordinary college students. Based on the ESP-MESH network, the lower computer realizes automatic networking between devices and tests the performance of the mesh network. Fourteen male college students received 9 weeks of agility training, with seven students in each of two groups: traditional agility training and agile equipment training. The researchers evaluated the performance of both groups in rapid disguise, body coordination, changing movements, and predictive decision-making.

**Results:**

There was no significant difference between the groups before training, but there were significant differences in the four abilities after training (*p* < 0.01). The experimental group had significant differences in rapid direction change and physical coordination (*p* < 0.05), and in changing movement and predictive decision-making ability (*p* < 0.01).

**Conclusion:**

Both traditional training and agile equipment training improve the agility quality of college students, and the latter shows better results in certain abilities. However, limited by other physical qualities, the improvement of motor changes and predictive decision-making ability is not as obvious as the other two abilities.

## 1. Introduction

In the field of sports, sensitivity refers to the ability of athletes to maintain body balance stably, control speed and strength well, change the direction of movement of the body in a coordinated manner, and accurately move to a reasonable spatial position when the sports environment around the training venue changes abruptly (Jin, 2020). Proper agility training can improve the agility and sprint speed of lateral movements and enable trainers to complete movements quickly during mobile defense (Forni et al., 2021). Good sensitivity has an important impact on ordinary students to establish correct technical motivation, master the techniques of various sports, and form good muscle proprioception (Yu, 2016). While developing sports qualities such as speed, strength, and endurance, athletes can increase the development of athletes’ sensitive qualities, so that they can more comprehensively improve athletes sports skills and physical fitness (Luo and Yang, 2020).

A wireless *ad hoc* network is a new form of the network compared to the current form, which does not require infrastructure support, and nodes form a multi-route wireless network through their own organization. It has the features of flexible networking, high compatibility, reliability, and high bandwidth (Jiang et al., 2021). The high adaptability of wireless *ad hoc* networks to application environments has led to the fact that the range of their applications has expanded to military, catastrophic, industrial, environmental, medical, and other capabilities. For example, special networks installed on vehicles (Ogundoyin and Kamil, 2021), coordinated operations of military multiplied aviation systems (Yuan et al., 2021), and research and development of wireless *ad hoc* network communication systems for rescue and disaster relief (Bi, 2019), etc. However, it has not yet been applied in the field of sports, therefore, the use of a Wireless *ad hoc* network for flexible learning can increase the reliability, invulnerability, and flexibility of the network.

The Internet of Things (IoT) is growing rapidly, with billions of smart devices being applied to objects to collect real-life information. However, the cost of hardware devices limits the data processing speed of smart devices, resulting in slow response, time delay, etc. (Wu et al., 2022b). The agile training of wireless *ad hoc* network has the characteristics of high response and low latency, which can better solve the problem of data processing.

At present, the agility training of ordinary colleges and universities still relies on mechanical equipment, such as hexagonal balls, soft ladders, standard cylinders, etc. (Ma, 2018; Wu, 2018; Zhang, 2019). FitLight, an electronic training equipment designed by Canada, is expensive, and the price of a set of equipment is as high as tens of thousands, which is not suitable for ordinary college training. Rusdiana (2021) studied an agile training instrument, although the randomness of training is achieved through hardware and software, it cannot measure the comprehensive response of user training. Wu (2015) researched an agile training instrument based on ZigBee technology, which cannot be networked between devices, and the training range is relatively limited. The training and testing methods for sensitive quality in relevant previous studies are relatively single, taking badminton as an example, there is a lack of research on sensitive quality in training special sports (Meng, 2022).

From a hardware and software architecture perspective, there is currently a lack of cost-effective sensing technology that can be easily integrated into manufacturing systems. Therefore, choosing the right sensor from among the few candidate sensors to maintain a balance between price and performance becomes an important task (Wu et al., 2022a).

For most of the current funny sports agile training has problems such as insufficient funds, and the use of traditional training methods and single training methods, the development of agile training instruments can promote the research on students’ sensitive quality, provide intelligent and affordable teaching equipment for college physical education, teachers can design and arrange more efficient and reasonable teaching plans, and improve the sensitive quality of college students. Therefore, it is necessary to develop an instrument suitable for agile training of wireless *ad hoc* networks for ordinary college students.

## 2. System design

**Figure 1:**
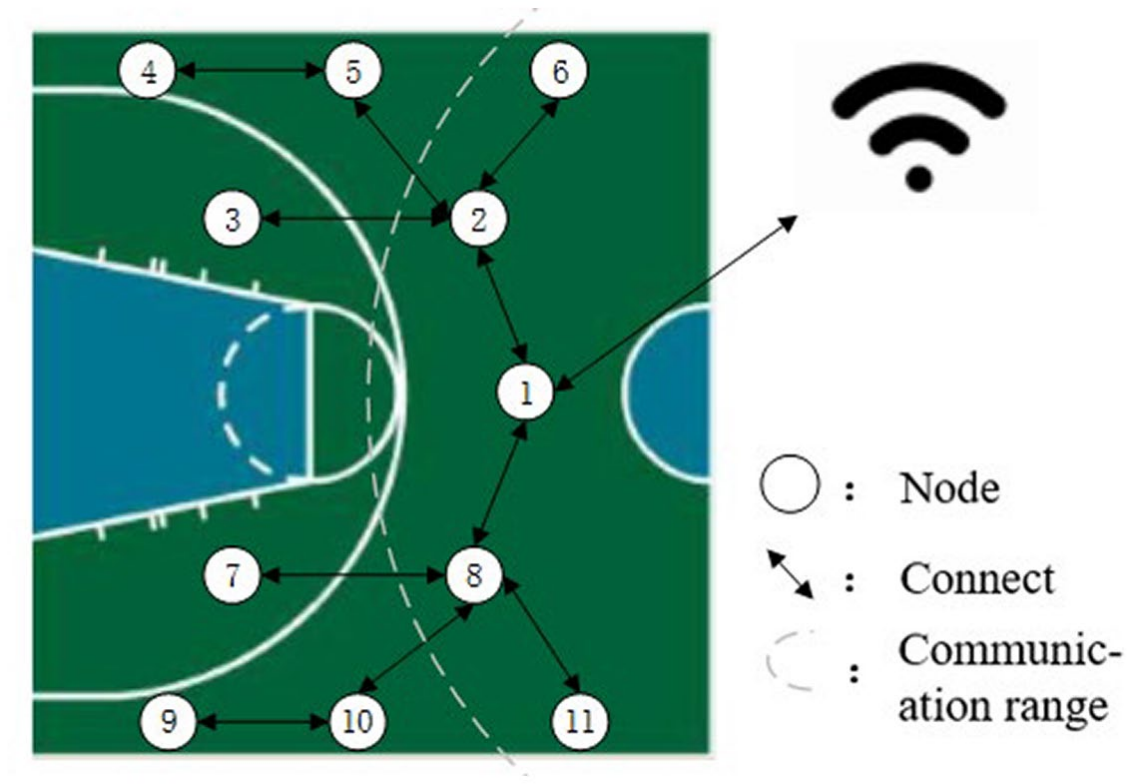
Schematic diagram of simulated random lower computer placement.

**Figure 2:**
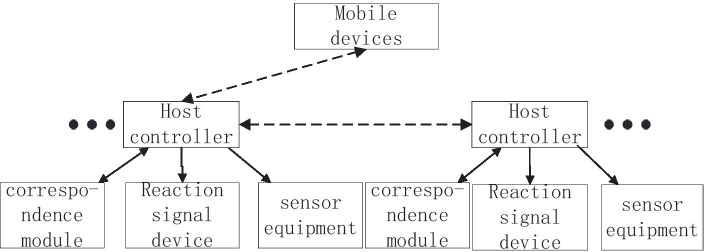
Overall system design diagram.

The system design idea is shown in Figure 1, taking the basketball court half court as an example. In the training preparation stage, the coach turns on all the required lower computers and randomly places it on the basketball court, the mobile device, and the lower computer are in the same LAN, and the athlete is in the center of the field when the athlete is ready, the coach sets the training times through the mobile device and sends the start command, the random lower computer will start the sound and light display to indicate that the point basketball will fall, and at the same time the lower computer starts timing in the main controller, the athlete quickly runs to the point to trigger the sensing device within the sensing distance, and the main controller stops timing and sends the time data to the mobile terminal, at which point a round is complete. Then the mobile terminal sends a start command to a certain lower computer point again, the sound and light display of the point will be activated, and the athlete will trigger the induction device again to complete the second round of agility training. Cycle through until you complete the number of pieces of the training set by the preparation phase. The overall scheme design of the system is shown in Figure 2.

### 2.1. Lower computer system design

Table 1 shows the structural parameters of the lower computer.

**Table 1:** Structural parameters of the lower computer of agile training instrument.

Structure	Parameter
lithium battery	Capacity: 1050 mA
buzzer	Audio intensity: 100–500 HZ
Light-emitting diodes	Luminous brightness: 80–90 Lm
PNP photoelectric sensor	Detection distance: 0–200 mm
Lower computer	Length × width × height: 7.0 cm × 5.1 cm × 4.2 cm Weight: 103.9 g

The hardware of the computer under the agile training instrument mainly includes the main controller, photoelectric sensor, ESP32 communication module (Lin, 2020), LED, and buzzer to realize signal monitoring, display, storage, and data transmission. The software includes an application program and a hardware driver, wherein the application implements data computing, signal control, and system networking, and the hardware driver is used to complete the setting of the working mode of the ESP32 communication module and the connection of the local area network. The power module is a 12 V lithium battery that can be charged and discharged. At the same time, different types of step-down switching voltage regulators are used, integrated circuit power modules are managed, and related voltage transfer circuits are designed to support the normal operation of different sensor modules (Xiao, 2021).

**Figure 3:**
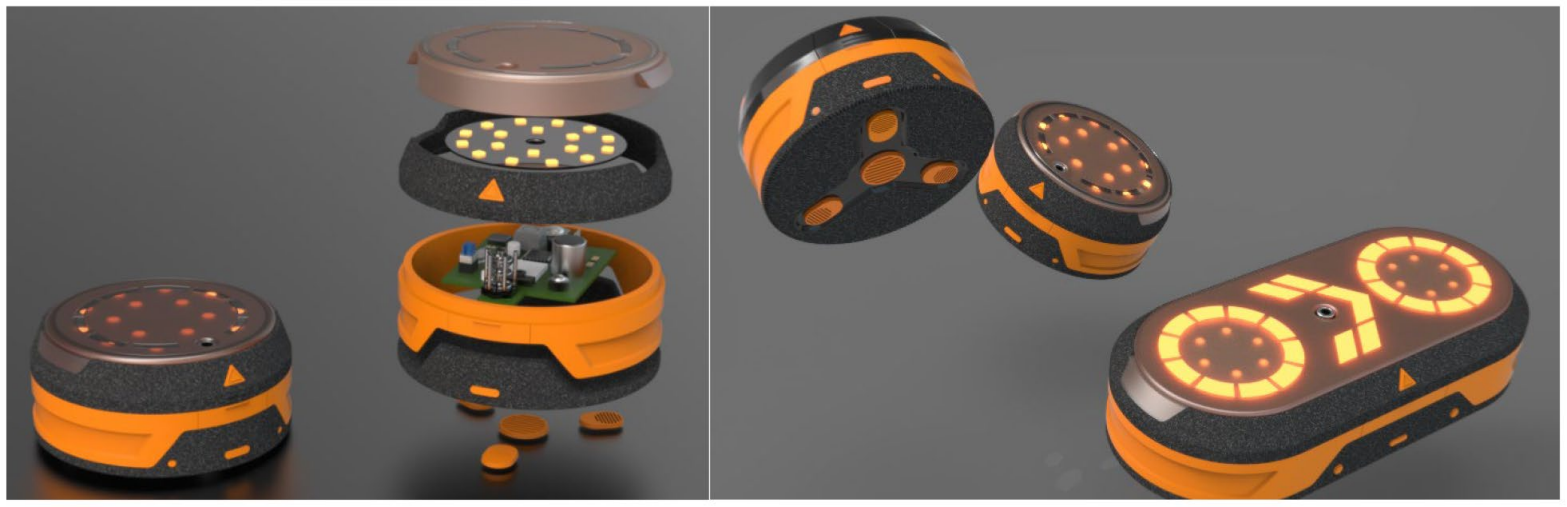
Lower computer rendering.

Figure 3 shows the effect diagram of the lower computer of the agile training instrument.

**Figure 4:**
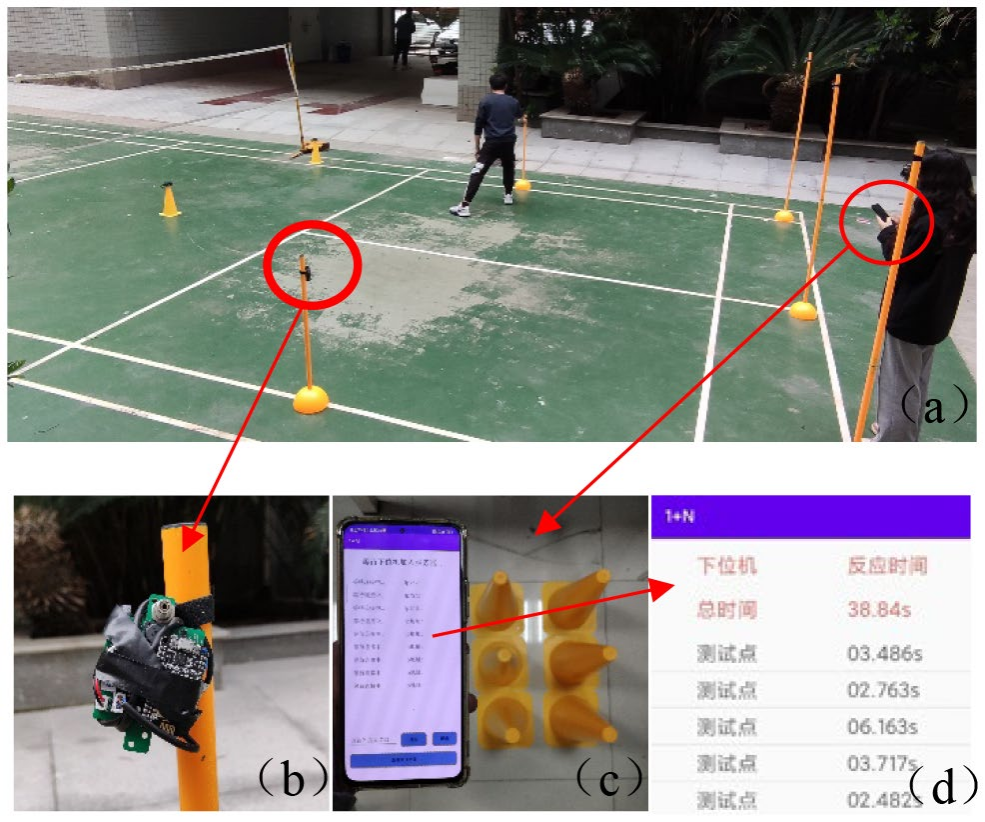
Agile training equipment. **(a)** training process, **(b)** lower computer, **(c)** upper computer application, **(d)** display interface.

Figure 4A shows the agile training process, (B) is the lower computer, (C) is the upper computer application, and (D) is the display interface.

### 2.2. Software design of host computer system

The host computer system application software program includes system communication, reaction training design, and UI interface design. In system communication, the upper computer needs to complete the establishment of the TCP server and listen to the connection request of the root node of the lower computer, and at the same time send the start command and accept the data during the movement. It is also necessary to complete the reaction training design according to the randomness of agile training and design the operation interface of humanization and data visualization.

#### 2.2.1. Communication system design

**Figure 5:**
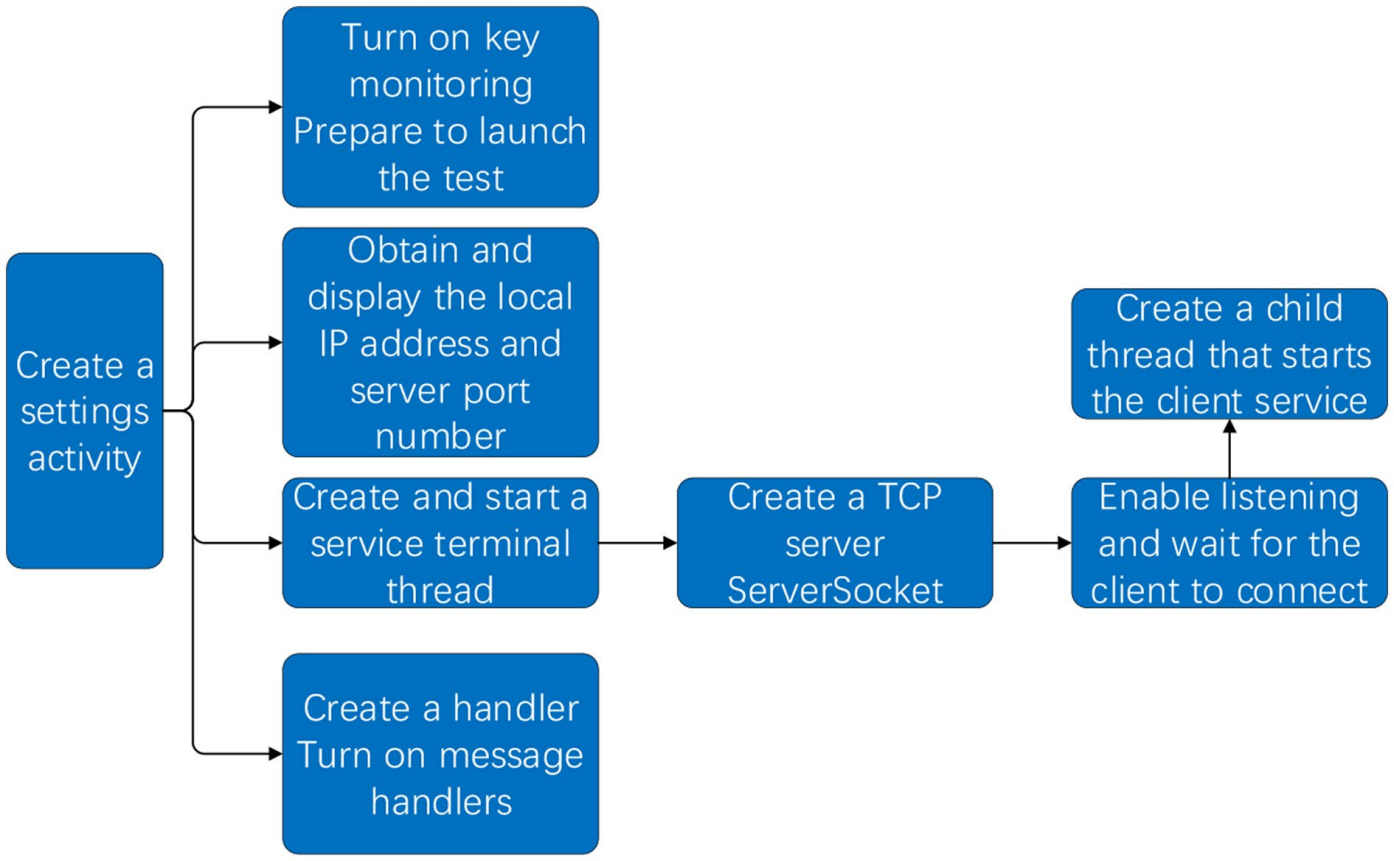
System wireless communication flow chart.

The host computer is designed using JAVA Socket technology to create and realize wireless connection and communication based on TCP/IP protocol. Android programs need to display the IP address of the machine in the LAN so that the client can connect to the specified IP address and port server. To complete the functional requirements of creating a TCP server, the program needs to use the ServerSocket class to create a server-side Socket, that is, the TCP server and open the connection that listens to the waiting client, and once there is a client connection, it is necessary to create a thread to process the connection between the client and the server to process the information interaction of the client separately. Use the WiFiManager class to obtain the information of hardware WIFI, and then obtain the local IP address and display it on the operation interface. In the function readFromClient, an attempt is made to read the client’s data from the input stream, and when the program catches an abnormal situation, it indicates that the client corresponding to the Socket has been closed, that is, the root node has lost communication with the host computer (Xiao, 2021). The system wireless communication flow diagram is shown in Figure 5.

#### 2.2.2. Training system design

**Figure 6:**
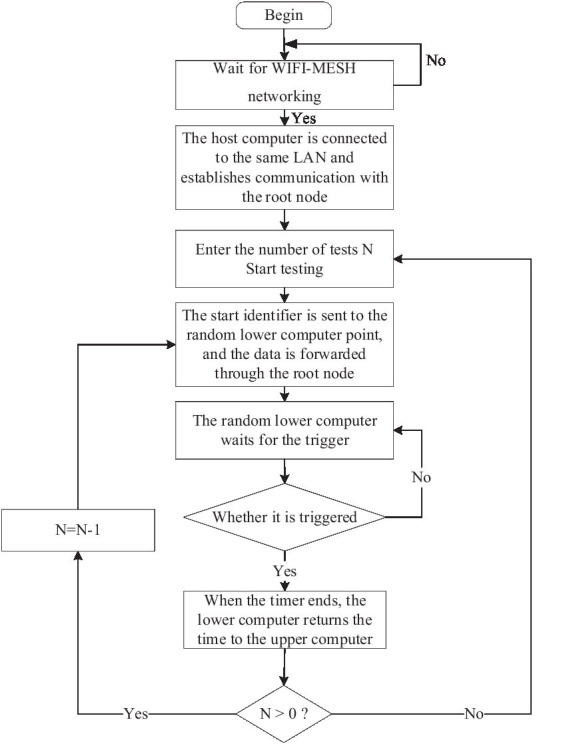
Upper computer design flowchart.

The design flowchart of the computer of the motion agility training system is shown in Figure 6.

According to the characteristics of the motion agility training system, after the upper computer waits for all touch front-end (lower computer) to complete the networking, the root node of the lower computer will obtain the MAC address of all child nodes and forward it to the upper computer, and the upper computer identifies the lower computer according to the unique MAC address and communicates with the lower computer through the root node. After entering the number of tests *N* through the host computer APP, click Start Test. The upper computer sends the start flag character to the random lower computer, the data is forwarded through the root node, and the main controller opens the timing interrupt and the serial port interrupt, waiting for the athlete to trigger, close the timing interrupt, and serial port interrupt, the main controller calculates the reaction time and returns to the upper computer, waiting for the athlete to trigger the lower computer, the lower computer returns the flag character and the sports data and views the comprehensive reaction through the mobile terminal (Wang, 2022).

### 2.3. Mesh networking

To improve network robustness, expand the training range. ESP32 module is used to realize networking function. ESP32-based MESH networks differ from traditional Wi-Fi networks in that the sensor devices in the network do not need to be all connected to the router, but can be connected to neighboring sensor devices. Each sensor device is responsible for the data relay of connected devices. Since it is not limited by the location of the router, as long as one sensor is connected to the router, the rest of the sensors can be connected within the communication range, so the expansion area of the mesh network is wider. At the same time, because it is no longer limited by the capacity limitation of router access, mesh networks allow more sensor devices to access and are not easy to overload (Xu, 2013).

#### 2.3.1. Networking software design

**Figure 7:**
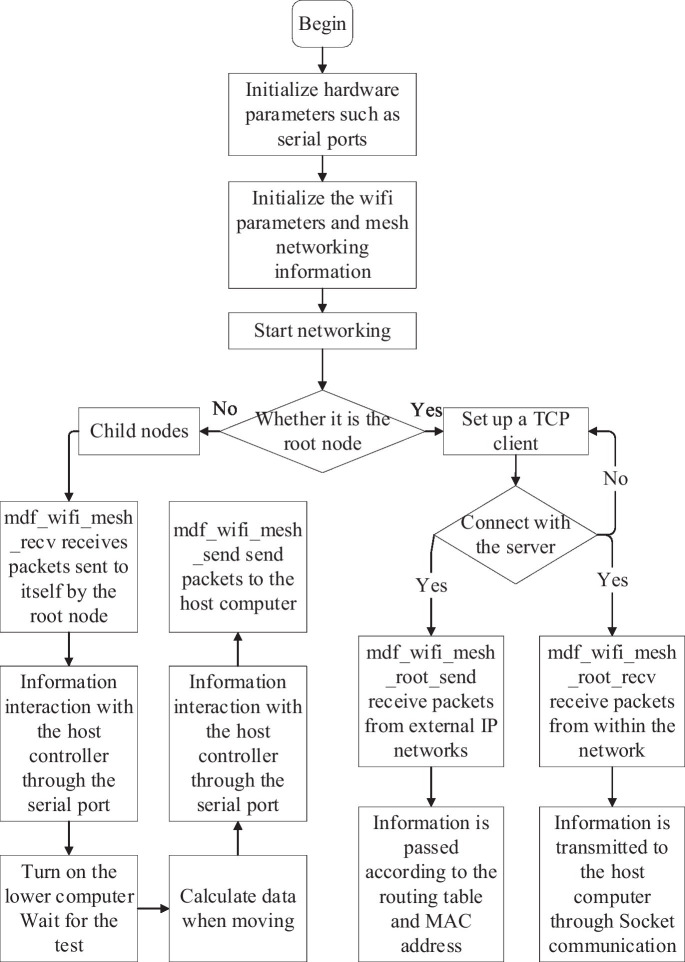
ESP32 software flow chart.

ESP-MESH initializes the stack required for non-volatile repositories and underlying TCP/IP before normal startup. Initialize ESP WiFi Mesh, register MESH event handlers, set the main parameters such as the maximum number of network layers, root node recommendation percentage, router name, password, and channel, etc., and finally call esp_mesh_start to start the MESH network (Li, 2019). The software design flowchart is shown in Figure 7.

When the node is the root node, create a TCP client, establish a TCP connection with the host computer, receive the opening instruction from the host computer, determine the lower computer, and send packets according to the routing table and destination MAC address stored in the root node. At the same time, it receives data from the network and sends the data during the motion to the host computer through Socket communication (Kuang et al., 2021).

When the node is not the root node, it will open the serial port when receiving information from the root node, interact with the host controller through the serial port, and complete the issuance of the opening command and the reception of data during movement.

#### 2.3.2. Network performance testing

The network networking performance was tested, mainly including networking time, new node joining the network, root node fault healing time, subnode fault healing time, node response time, and packet loss rate (Ni et al., 2021). The test results are shown in Table 2.

**Table 2:** Performance parameters related to mesh networks.

Performance	Time
Network construction time	8 s–15 s
Node join time	<10 s
Root node failure repair time	<10 s
Child node failure repair time	<5 s
Node response time (three layers)	280 ms–350 ms
Packet loss rate (within three layers)	<1%

Under MESH network networking, excessive network layers will lead to an increase in the number of data forwards, longer response times, and ultimately a decrease in communication quality. The number of computer devices under the agile training system is generally less than 15, which is limited by the range of the sports field, the number of network layers will not exceed three layers, and the response time of node forwarding data is less than 350 ms, so it will not affect training.

## 3. Research objects and methods

### 3.1. Research object

To verify the effectiveness of agile training instruments in the sensitive quality training of college sports students, 14 students were selected as experimental subjects according to the physical education test data with the Hubei University of Technology as the research site. According to their different training methods, they are divided into traditional groups and experimental groups. Among them, there were 7 people in the traditional group and 7 in the experimental group. All subjects are aware of the purpose, process, possible risks, and precautions of this test. One week before the test, none of the subjects were injured or subjected to an intense training load. According to the results of the data in Table 3, the relevant physical statistics *p* > 0.05 indicate that the correlation of the two groups of experimental subjects is similar, and there is no significant difference between the experimental group and the control group in terms of relevant physical state and sensitive quality.

**Table 3:** Data table of physical fitness of experimental subjects.

	Traditional group (*n* = 7)	Experimental group (*n* = 7)	*p*-value
Age/years	21.50 ± 1.21	21.25 ± 1.28	0.745
Height/cm	179.57 ± 1.6	180.01 ± 3.3	0.580
Weight/kg	70.74 ± 1.88	69.23 ± 1.37	0.136
Body Fat %	15.38 ± 0.38	15.51 ± 0.29	0.535
BMI (kg·m^−2^)	21.96 ± 0.97	21.27 ± 0.58	0.162
Pro Agile test/s	5.22 ± 0.52	5.12 ± 0.19	0.642
Hexagonal test/s	7.88 ± 0.61	7.61 ± 0.58	0.227
*T*-run/s	12.01 ± 0.41	11.90 ± 0.58	0.184
5 m three-way switchback test/s	9.35 ± 0.16	9.49 ± 0.28	0.293
505 in disguise test/s	5.52 ± 0.37	5.36 ± 0.29	0.247
15 s stand-ups/pcs	5.62 ± 0.42	5.57 ± 0.43	0.848

### 3.2. Research methods

The traditional group and the experimental group each conducted 9 weeks, 4 training sessions per week, each training session of 30 min of agility training. The traditional group conducts traditional agile training in stages, and the experimental group trains with the help of agile training instruments. By comparing the four test data of the traditional group and the experimental group after training, it is verified that the agile training instrument designed in this paper has a practical effect on the improvement of sensitive quality. In order to ensure that the data obtained are true and valid, athletes take an average of each test indicator after three tests. SPSS 21.0 was used for data statistics, expressed by mean number ± standard deviation, and independent samples were tested for four indicators before and after the experiment, and the differences between the groups were expressed by *p*-value, and *p* < 0.05 indicated that there was a significant difference.

Traditional group training is divided into three stages: before, during and after the stage, and the specific training arrangement of each stage training is shown in Table 4 (Pan, 2018; Zhang and Dai, 2022; Tan, 2023).

**Table 4:** Training schedule of traditional group.

Training phase	Training content	Training purpose	Training duration	Training intervals
Pre-stages	1. Slide backwards	Understand basic agility training to ensure quick completion of movements and establish basic movement patterns	1–2 weeks 3 sets at a time Each set of training is about 10 min	1–2 min
	2. Sit and clap your hands to stand			
	3. The sideslide step bottomed out 4. Small broken steps, etc.			
Medium phase	1. Hexagonal training	On the basis of quickly completing movements, practice directional training, movement changes and predictive power	3–5 weeks 3 sets at a time Each set of training is about 12 min	2–3 min
	2. 505 Training in disguise			
	3. *T*-run			
	4. Illinois Sensitive Run, etc			
Later stages	1. Rope ladder training	Combined with mechanical equipment, improve the explosiveness and coordination of the body, and further improve the sensitivity quality	5–9 weeks 3 sets at a time Each set of training is about 15 min	3–5 min
	2. Marker tube training			
	3. Hurdle training			
	4. Agility circle training, etc.			

**Figure 8:**
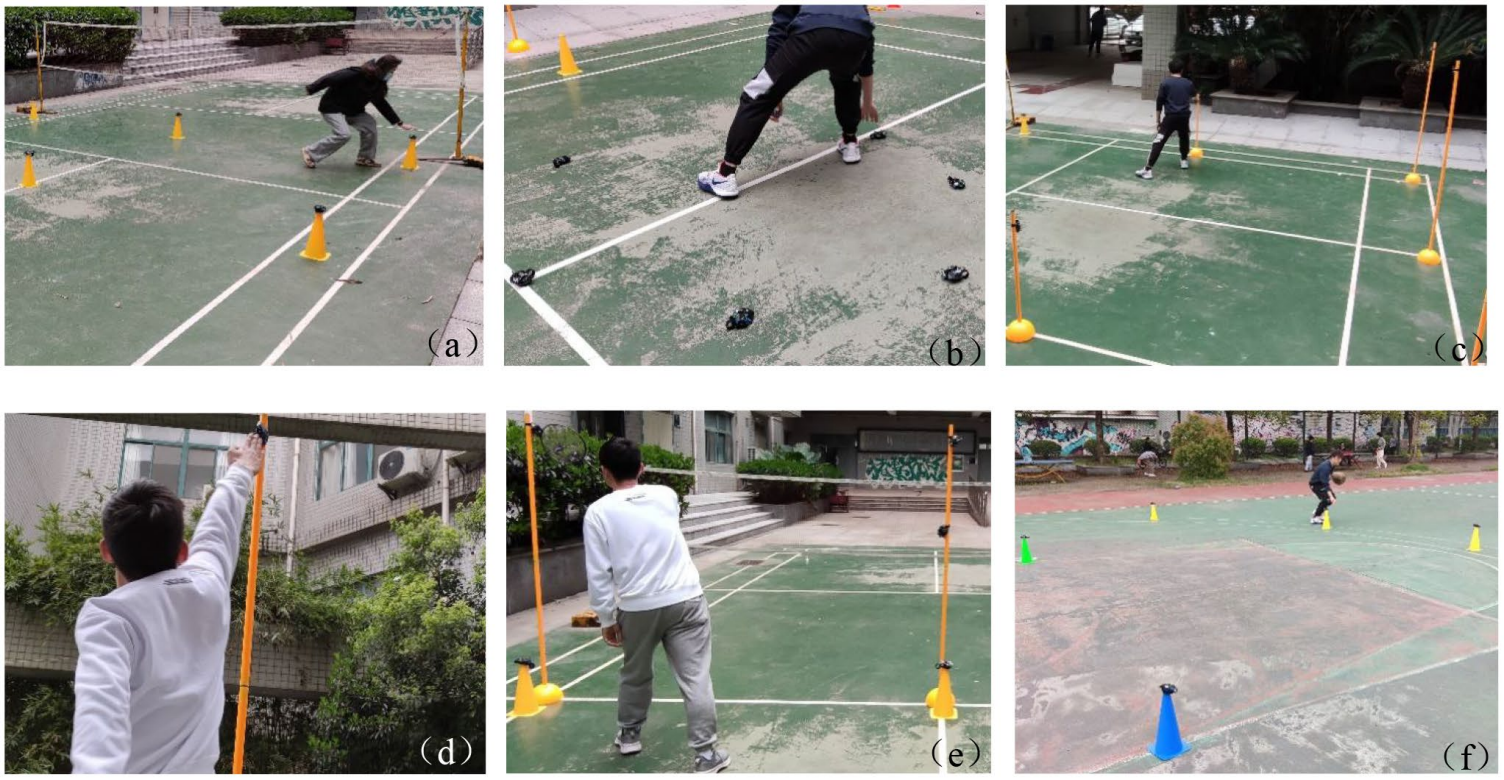
Relevant training content diagram of the experimental group. **(a)**, **(b)** warm-up phase, **(c)**, **(d)** strengthening phase, **(e)**, **(f)** training phase.

The training of the experimental group was divided into a warm-up phase and an intensive phase. By asking experts in physiology and physical training, the agile training content of the experimental group was designed in a targeted manner, and several training actions were formulated with the help of agile training instruments and related mechanical equipment. The warm-up phase includes frontal touch instrument training, hand and foot touch training, random placement touch training, and other agility training. This is shown in Figures 8A,B. The strengthening stage includes hand and foot touch equipment training, random re-entry run touch training, and random touch training, as shown in Figures 8C,D. According to the different sports characteristics of the sports learned by the experimental subjects, the agile training equipment in different positions was placed, and a variety of training methods were designed for special training. As shown in Figure 8E, taking badminton as an example, nine test points of the lower computer are placed on the field to mimic the position of the opponent’s hit. At the same time, with the help of metal brackets, the agile training equipment is placed in random combinations of different heights to simulate six types of technical response scenarios (rubbing the ball, hooking the ball, pushing the ball, hanging the ball, killing the ball, high distance ball). As shown in Figure 8F, taking basketball as an example, about 15 pieces of agile training equipment are randomly placed in the basketball half, and the trainer touches the random agile equipment while holding the ball to achieve the effect of improving its agility, and with the help of networking technology, the control of all agile instruments can be realized.

Zhao et al. (2014a,b) believe that the sensitive quality model of college students is composed of three factors: the ability to change direction, the ability to change movement and coordination and the ratio of factor load of the three is 77:91:89. The relevant agile test factors such as Illinois training and cross change running in changing direction are 69:83, standing up, standing up and sitting clapping are 52:50:71 in changing movements, and “hand dancing” and repeated spans are 80:46 for coordination. Stewart et al. (2014) tested the validity of five commonly used CODS tests and showed that they all had low percentage error and high relative reliability. Zhao (2011) shows that standing up is a test index of sensitive quality, including the ability to change direction and change movements. Yan (2021) through research, the hexagonal ball test can well reflect the predictive decision-making ability of athletes. Therefore, this paper divides motor sensitivity into predictive decision-making ability, change movement, body coordination ability, and rapid direction change ability.

Comprehensively consider all factors such as the relative reliability (ICC) of different sensitive test solutions, the ability to be single and highly representative of a specific sensitive quality, and the actual test site. The four test methods of 5 m switchback running, 20 s repeated cross-jump, 15 s stand-up and hexagonal ball reaction ball were selected to reflect the subjects’ rapid change of direction, body coordination, change of movement and predictive decision-making ability. Some of the sensitive aptitude test schemes are shown in Table 5.

**Table 5:** Sensitive test scheme.

Test the content	Sti-mulate	Test distance (m)	Time (s)	ICC
Illinois training	No	60	15–20	0.89
*L*-run	No	20	5.5–6.5	0.94
Pro-Agility	No	18–19	4.9–5.3	0.90
*T*-run	No	About 42	7.5–10	0.95
505 sensitivity test	No	10	2.4–2.6	0.85
Three-way switchback run	No	15	7.5–10.5	0.94
*Y*-test	No	10	1.8–2.1	0.88
Repeated sideways	No	3	20	0.86
93,639 turned around	No	33	7–9	0.95
Straight sprint	No	10	2–3	0.86
Direction change test	No	8–9	3–4	0.86
Stand-ups	No	1–2	15	0.95
Hex ball reaction test	No	2	2.7–3.9	0.95

The specific rules for the four training methods for testing are as follows:

1. 5 m three-way switchback run.

**Figure 9:**
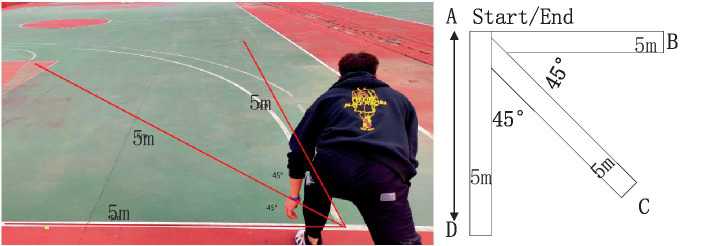
Schematic diagram of 5 m three-way switchback run.

Test method: as shown in Figure 9, the use of tape to paste three 5 m long straight lines, the tester heard the password and started, the tester began to clock at the same time, and the tester ran from the starting point A to point B at 5 m with his hand to touch the retracement line and return to point A, and then to point C, point D and finally return to point A, the time spent was the final score. This testing scheme evaluates the ability to change direction quickly by using rapid braking and steering.

1. 20 s to repeat the jump.

**Figure 10:**
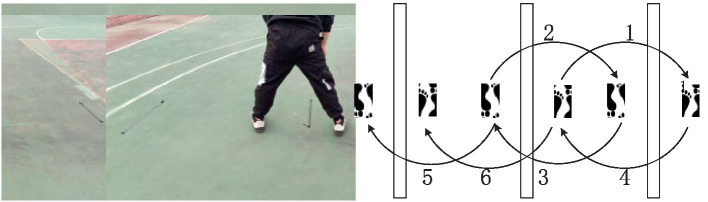
Schematic diagram of 20 s repeated span jump.

Test method: as shown in Figure 10, use tape to paste three parallel lines with a distance of 1 meter, the tester stands with both feet across the midline, knees slightly bent, and after hearing the gun password, crosses the right line to the right, and then cross the midline to the left; Then cross the left line to the left, and so on, press “1–6” as a group of repeated jumps, and record the number of crossings in 20 s. The project is tested three times, and the average of the three times is taken as the final grade. This test protocol evaluates physical coordination by how fast or slow the feet are constantly moving.

1. Hex reaction training.

Test method: the tester places the hexagonal reaction ball in a 2 meter-high position, the tester stands 1.7 meters in front of the projection point of the hexagonal reaction ball, allows the hexagonal ball to fall freely, and catches the ball at the fastest speed when the hexagonal reaction ball lands and bounces. Calculate the time it takes for a player to go from falling the ball to catching it. This testing scheme evaluates the predictive decision-making ability by determining the position of the hexagonal ball bounce.

1. 15 s stand-ups.

**Figure 11:**
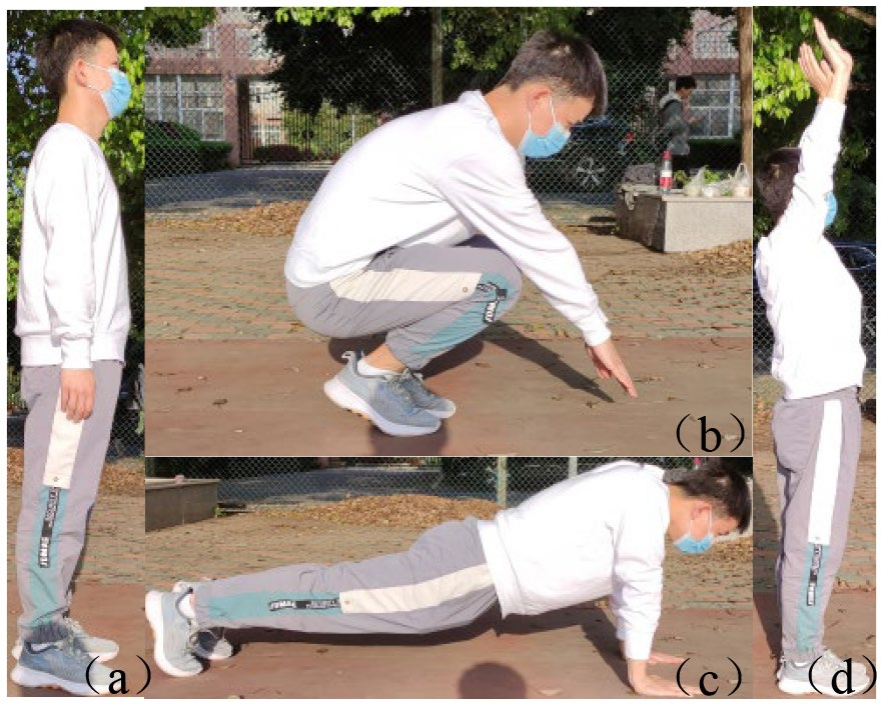
Schematic diagram of 15 s stand-ups. The sequence of **(a–c)**, and **(d)** is a stand-up action.

Test method: as shown in Figure 11A, the tester stands with both feet horizontally, after hearing the gun password, quickly bends his knees and bends, moves his hips back, squats down, and supports his hands at the distance of one palm in front of his feet, and the lower limbs are extended back at the same time to make the body into a push-up position, complete a push-up, and then withdraw the lower limbs to the abdomen to squat in a deep posture, and finally stand upright. Repeat until the end of the 15 s. Figures 11A–D is ordered by one stand-up action. This protocol evaluates rapid change of motion by rapidly changing position.

## 4. Experimental results and analysis

After the training of the traditional group and the experimental group, the results of the sensitive quality and multiple ability data of the experimental subjects are shown in Table 6.

**Table 6:** Comparison table of pre/after training data between traditional group and experimental group (*n* = 14).

Constituencies	Serial number	Test: 1 (sec)	2 (pcs)	3 (pcs)	4 (sec)
Traditional group	1	9.51/8.84	5.33/6.67	5.33/7.33	3.53/2.96
	2	9.47/9.01	5.67/7.00	5.00/7.00	3.45/2.94
	3	9.05/8.73	6.33/7.00	5.67/7.67	3.43/2.83
	4	9.40/8.82	5.67/6.67	5.67/7.67	3.35/2.83
	5	9.22/8.91	5.67/6.67	6.00/8.00	3.31/2.98
	6	9.30/8.83	5.33/6.33	6.33/8.33	3.77/3.19
	7	9.50/9.06	6.67/7.67	5.33/7.33	3.30/2.73
Experimental group	1	9.13/8.77	6.00/8.00	5.33/8.00	3.51/2.50
	2	9.66/8.70	6.33/7.67	5.33/8.00	3.69/2.72
	3	9.15/8.36	5.00/7.33	6.00/8.33	3.41/2.50
	4	9.27/8.68	5.67/7.33	5.67/8.33	3.64/2.83
	5	9.75/8.79	5.33/7.33	5.33/8.00	3.79/2.95
	6	9.79/8.53	5.67/7.00	5.00/7.67	3.34/2.44
	7	9.72/8.71	7.00/8.00	6.33/8.67	3.59/2.78

Test 1 is a 5 m three-way switchback run; test 2 is a 20 s repeated span jump; test 3 is a 15 s stand-up; test 4 is a hexagonal ball reaction test.

### 4.1. Comparative analysis of data before and after traditional group experiments

**Figure 12:**
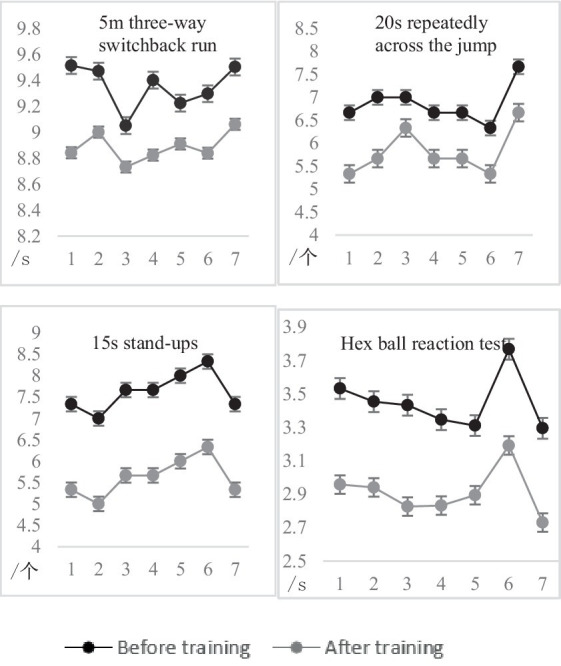
Comparison of the test results of the traditional group before and after the experiment.

To study the fluctuation degree of sensitive quality of experimental subjects in the traditional group before and after traditional training, the sample *t*-test was used to compare and analyze the sensitive quality ability data of the experimental subjects, and the results are shown in Figure 12 and Table 7.

**Table 7:** Comparison table of pre/after training data of traditional group (*n* = 14).

Test the project	Before training	After training	*p*-value
5 m three-way switchback run (s)	9.35 ± 0.16	8.87 ± 0.10	^**^
20 s span jump (pcs)	5.81 ± 0.47	6.86 ± 0.39	^**^
15 s stand-up (pcs)	5.62 ± 0.42	7.62 ± 0.42	^**^
Hex ball response test (s)	3.45 ± 0.15	2.91 ± 0.14	^**^

^*^Indicates that the difference between the test items of the traditional group and the experimental group is significant, 0.01 < *p* < 0.05; ^**^indicates that the difference between the test items of the traditional group and the experimental group is very significant, 0.00 < *p* < 0.01, the same below.

**Figure 13:**
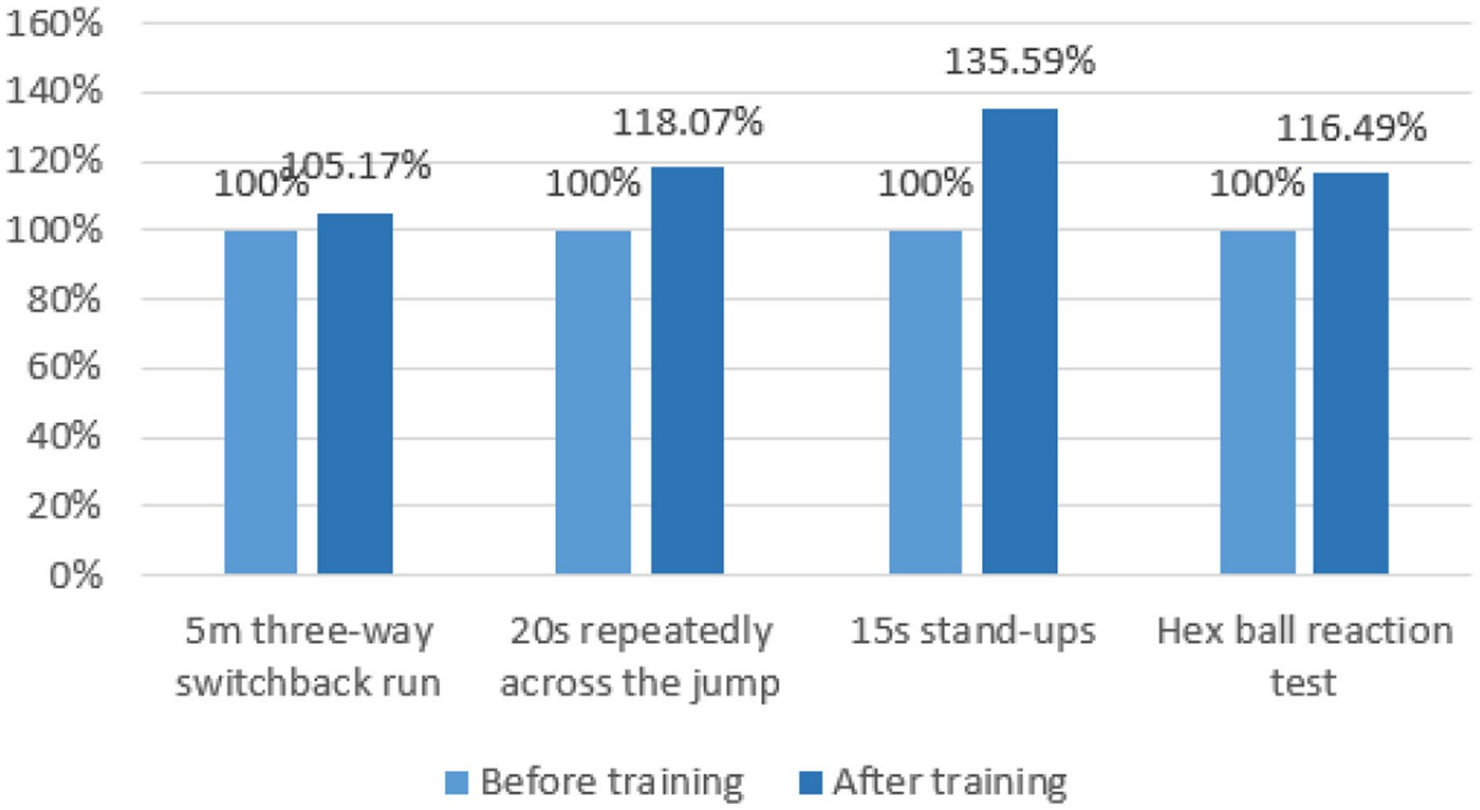
Growth rate of various test scores in the traditional group before and after the experiment.

The paired-sample *t*-test, *p* < 0.01, and the data of the two groups of each test showed a very significant difference, indicating that the performance of each test was improved to a certain extent. After training, it can be seen from Figure 13 that the results of the traditional group increased by 5.17%, 18.07%, 35.59%, and 16.49%, respectively, compared with before training. Therefore, it is explained that through traditional training, the tester’s ability to change direction quickly, body coordination, change movements and predict decision-making ability can be improved.

In addition to the strength of the lower limbs, the main effects affecting the ability to change direction and change movements are the reaction force during braking, the fast and slow cadence when moving, the size of the pace, and the excitation and inhibition conversion speed of the central nervous system. Therefore, improving the excitability of the nervous system, establishing information transmission channels between muscles and nerves, and improving the control ability of nerves over muscle movement play an important role in improving rapid direction change and change action ability (Chen et al., 2021). The *T*-type running and 505 direction change training in traditional group training not only stimulate the muscles to involve more motor neurons, improve the explosiveness and flexibility of the muscles, but also enhance the excitation and inhibition function of the brain nerves, improve the body’s control of the body’s emergency stop ability during high-speed travel, and better carry out body direction change and body coordination. Some scholars also believe that the training method with the help of mechanical equipment such as soft ladders improves the ability of each muscle in the motor chain to contract together, controls the proportion of anti-muscle contraction, controls the cooperative movement of flexor and extensor muscles, adductor muscles, internal rotation, and external rotator muscles, and reflex muscles, and the cooperative movement of upper limbs, trunk, and lower limb muscles, to better improve body coordination (Dong, 2013). França et al. (2022) conducted stretch compound training on athletes and analyzed their sports performance, and experimental data proved that there is a certain correlation between lower limb explosiveness and sensitive quality, and the stronger the explosive power, the higher the sensitivity quality of athletes. Chuang et al. (2022) demonstrated that proper agility training can improve the agility and sprint speed of lateral movements by performing 6 weeks of agility training on experimental subjects, and enable athletes to quickly complete movements during the mobile defense. Therefore, when training in the traditional group, these two abilities will be improved to a certain extent. The resultant force point of gravity in all links of the human body is called the center of gravity, including the center of gravity of the head and torso, and the change of human posture is an important factor restricting the center of gravity (Lu, 2021). Stand-up is a sport in which the center of gravity of the body changes rapidly during exercise, and there are high requirements for the coordination of athletes’ body movements. In traditional training, the exercises on the movement of the center of gravity are involved both straight and horizontal movements, and these exercises require the athlete’s center of gravity to be stable. Therefore, the ability to change movements will be improved to a certain extent. Agility circle reaction training uses the placement of different agile circles to carry out a variety of exercises, requiring practitioners to pay high attention, react quickly, start, make corresponding actions, and even predict in advance to ensure that they step into the correct agile circle. Chen (2021) by training the experimental group for 6 weeks of agility circles, SPSS statistical analysis was used before and after training, and it was concluded that the agile circle had a certain improvement in the ability of predictive decision-making (*p* < 0.01). This is the same conclusion as the experimental data in this paper. The 20 s reactive cross-and-hexagonal ball reaction test requires the tester to have a high reaction decision-making ability. In traditional training, hurdle, and agility circle training can stimulate the cerebral cortex, excite the nervous system and inhibit continuous conversion, improve the comprehensive analysis ability of the brain, and combine the tester’s muscles, footwork, and nervous system training to improve efficiency and enhance reflexes, thereby improving performance.

### 4.2. Comparative analysis of data before and after the experiment in the experimental group

**Figure 14:**
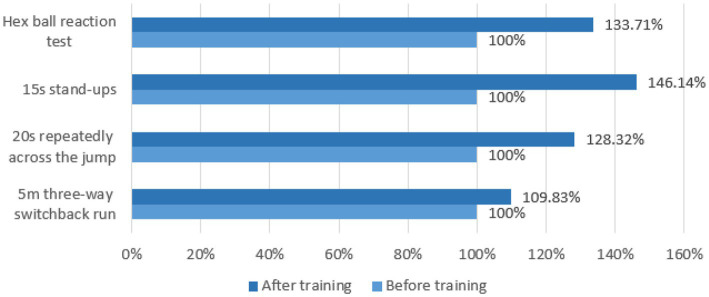
Growth rate of test scores in the experimental group before and after the experiment.

To study the fluctuation degree of sensitive quality of experimental subjects in the experimental group before and after training with the help of agile training equipment, the sample *t*-test was used to compare and analyze the sensitive quality data of experimental subjects, and the results are shown in Figure 14 and Table 8.

**Table 8:** Comparison table of data results before and after experimental group (*n* = 14).

Test the project	Before training	After training	*p*-value
5 m three-way switchback run (s)	9.49 ± 0.28	8.64 ± 0.14	^**^
20 s span jump (pcs)	5.86 ± 0.61	7.52 ± 0.35	^**^
15 s stand-up (pcs)	5.57 ± 0.42	8.14 ± 0.30	^**^
Hex ball response test (s)	3.57 ± 0.15	2.67 ± 0.18	^**^

Through the paired-sample *t*-test, *p* < 0.01 showed significant differences between the two sets of data in each test, and the results were improved after training. Therefore, it is explained that training through the agile training equipment developed in this design can improve the tester’s sensitive quality ability.

**Figure 15:**
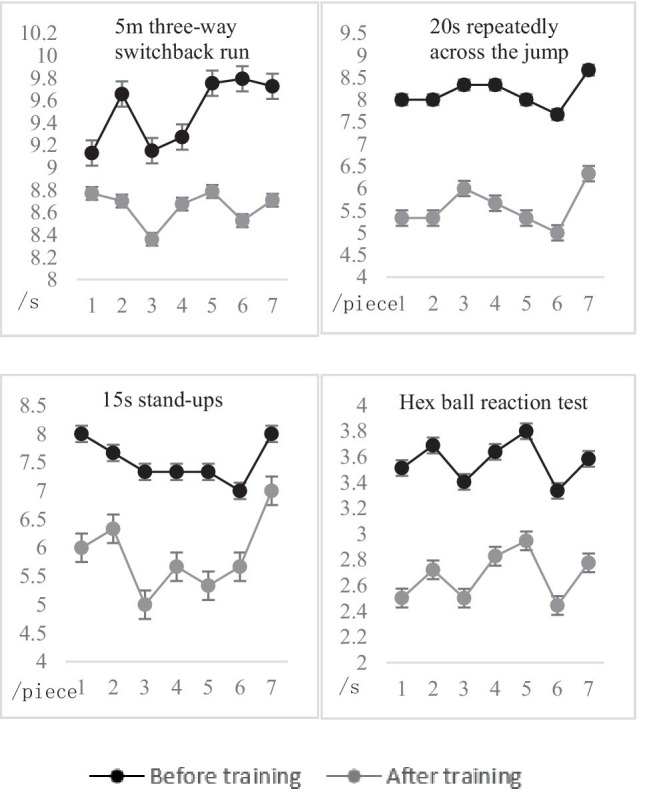
Comparison of test results of experimental groups before and after the experiment.

After training, it can be seen from Figure 14 that the results of the experimental group increased by 9.83%, 28.32%, 46.14%, and 33.71%, respectively, compared with before training. Agility training instrument training concerning traditional training methods based on adding randomness, the trainer’s nervous system is highly matched with physical activity, the hand, eyes, and brain should be flexible, and the trainer’s response ability can be effectively improved by continuing this high-intensity stimulation. The random placement and random signal of agile training equipment can realize training of different distances, different heights, and different sports methods, and at the same time combine the sports characteristics of different sports to achieve different needs (see Figure 15).

Short-distance random placement point training, hand and foot touch training, etc. in agile training can enhance the tester’s limb stability, coordination between muscles, and control ability between nerves and muscles, thereby improving the tester’s predictive decision-making and physical coordination. Therefore, after training, the test results of the experimental subjects will be improved in the 20 s repeated span and hexagonal ball test. Zemková and Hamar (2010) designed 6 weeks agile balance response training with the help of agile training instruments have similarities with the training content of agile training instruments, both are comprehensive training of a variety of agile abilities, and the results show that the combination of agility and balance training improves dynamic balance under visual control, improves muscle contractility ability, and better improves agility. Zech et al. (2010) has also experimentally concluded that agility and balance training is effective in improving posture and neuro-muscle control.

Long-distance random placement point training, random switchback running training, and random height training are training that combines speed, body disguise and coordination, and reaction ability to improve the coordination and movement effect of muscles in each link of athletes’ movement process, and help train the speed and power of body coordination under braking and cushioning conditions in different ways. Therefore, after training, the test subject’s 5 m three-way switchback test performance will be improved.

Training according to the characteristics of different sports can transform complex body movements into regular behavior, the brain controls muscles, joints, and bones to complete the established action procedures, strengthen muscle memory, and flexible joints, enhance foot movement efficiency, and better control the whole body force and coordination. Therefore, after training, the test subjects’ 15 s bench-up test scores will be improved. Dorothy and Martha (2013) tested agility before and after volleyball teaching and proved that training combined with sports has a good effect on agility improvement. Rogozhnikov et al. (2020) used FitLight for basketball training, and the results showed that the number of mistakes made by athletes when dribbling the ball was reduced by almost half, about 43.6%. This is mainly because the use of agile training instruments can improve not only neuromuscular connections but also the sense of touch of athletes.

### 4.3. Comparison and analysis of data after experiments between traditional group and experimental group

**Figure 16:**
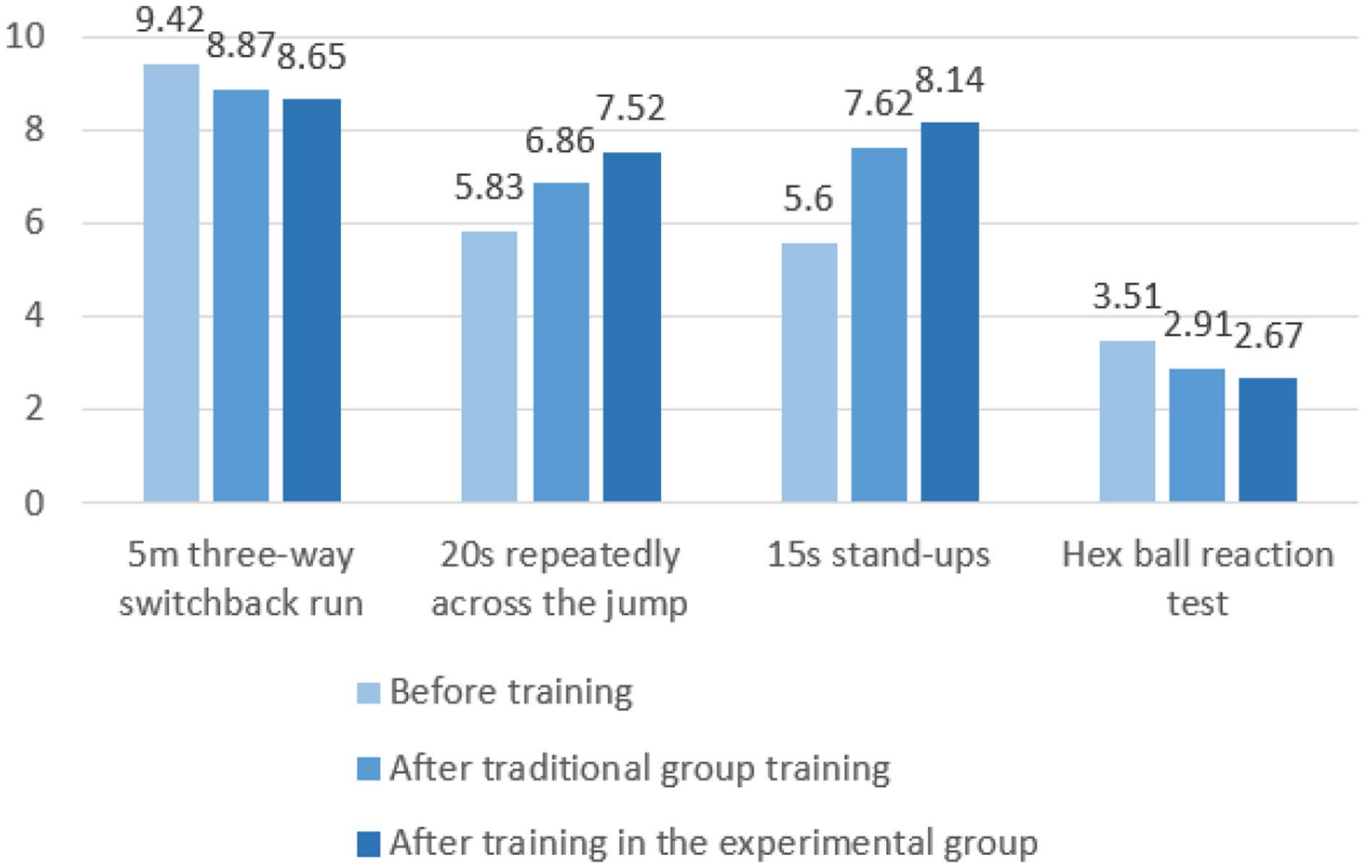
Comparison of test results between traditional group and experimental group before and after the experiment.

To study the difference between the traditional group and the experimental group after training, the sample *t*-test was used to compare and analyze the sensitive quality ability data of the experimental subjects, and the results are shown in Figure 16 and Table 9.

**Table 9:** Comparison of traditional group and experimental group after training (*n* = 14).

Test the project	Before training	After training	*p*-value
5 m three-way switchback run (sec)	8.89 ± 0.10	8.64 ± 0.14	^**^
20 s span jump (pcs)	6.86 ± 0.40	7.52 ± 0.35	^**^
15 s stand-up (pcs)	7.62 ± 0.42	8.14 ± 0.30	^*^
Hex Ball Response test (sec)	2.91 ± 0.14	2.67 ± 0.18	^**^

From Table 9, it can be concluded that after 9 weeks of training with agile training equipment, it was found that the *p*-value of the 5 m three-way switchback run and 20 s span jump was less than 0.01 by comparing the agile training results of the traditional group, indicating that the difference between the two test data was very significant, and the *p*-value of the 15 s stand-up and hexagonal ball reaction test was less than 0.05, indicating that the two test data were significantly different.

**Figure 17:**
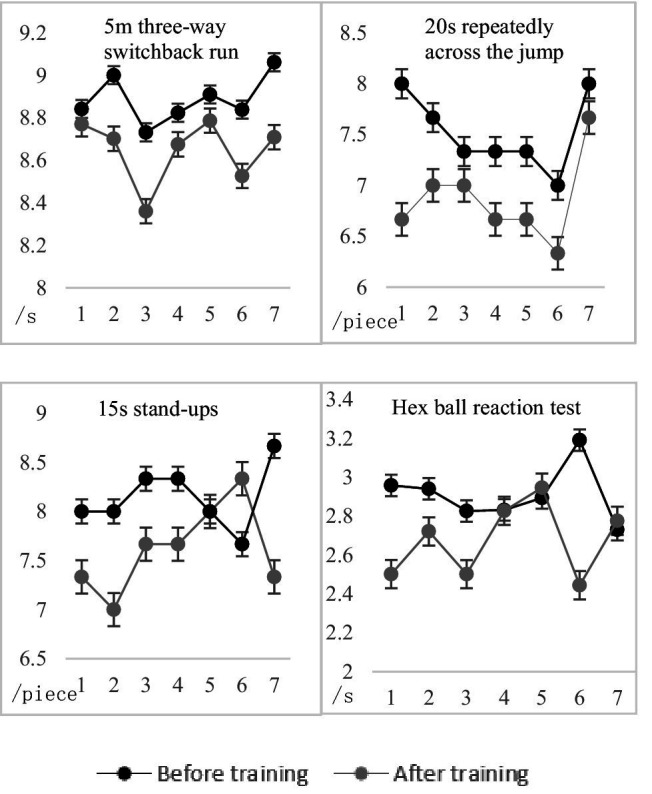
Comparison of the mean test scores of the two groups after training.

From the comparison of the average test results of the two groups after training in Figures 16, 17, it can be seen that the performance of the experimental group after training has improved compared with the traditional group. Random long-distance training and random switchback running training in the training content of the experimental group can fully stimulate the lower limb muscles during the training process, improve the muscle strength and flexibility of the knee, hip, and ankle, and improve the athlete’s ability to change movements and quickly change direction (Liao, 2021). Compared with the traditional training method, the reaction function of the agile training instrument can find the disadvantageous orientation in the rapid change of direction of the experimental subject and carry out intensive training for the weak link. Therefore, training with agile training instruments has a better improvement effect on experimental subjects than traditional training methods. Random short-distance training, frontal hand and foot touch training, and training combined with different sports in the training content of the experimental group can fully activate the brain nerves and multiple levels of skeletal muscles, coordinate the cooperation of upper and lower limbs, and enhance the explosiveness of the limbs (Wu, 2021). The random signal function can stimulate the functional state of the cerebral cortex and visual nervous system so that the test subject’s nervous system can better recruit motor neurons. Therefore, compared with traditional training, the physical coordination ability and predictive decision-making ability of experimental subjects can be better improved. In the 15 s stand-up and hexagonal ball reaction test in the test project, the *p*-value is less than 0.05, indicating that after 9 weeks of sensitive quality training, the experimental group is not very obvious in the ability to change actions and predict decision-making compared with the traditional group, but it also has a certain effect, because it involves the performance of the core strength of the human body and the neural response system, so it has higher requirements for the experimental subject than other abilities. Zhao et al. (2014b) used the repeated cross, cross jump test, stand-up, and round trip running as the test indicators of sensitive quality (including direction change ability and change movement ability), and pointed out that after 3 weeks of training, all four indicators were improved, only the standing up test performance did not reach a significant difference, pointing out that changes in body composition and resting metabolic rate had an impact on the sensitive quality of athletes. SAQ (speed, agility, quickness) training emphasizes the cultivation of athletes’ speed, agility, and quick responses. Ji and Cao (2022) proposed that adding SAQ training on the basis of traditional training can make the physical fitness training of volleyball players more systematic, professional and comprehensive, further expand the special mobility ability of volleyball players, help volleyball players improve speed, agility, rapid reaction ability and special movement ability, and improve the technical and tactical level and competitive level of volleyball players. After (Lin, 2021) used the agility ladder for special agility training, the experimental group of female basketball players in the basketball court “back” foot sensitivity test and restricted area “X” foot sensitivity test results were generally better than the traditional group. It showed that the sliding movement ability of the women’s basketball players after intensive training through the agility ladder was greatly improved, which had a positive effect on running without the ball and the acceleration of defensive footsteps. The agile training instrument of wireless *ad hoc* network not only has the advantages of SAQ training and ordinary special agile training, but also has the characteristics of not being limited by venue, diversified agile training and high sensitivity.

## 5. Conclusion

In this paper, a distributed badminton agility training device based on an embedded system and Android program is designed, which can realize functions such as free distribution, wireless connection and communication, and real-time display of comprehensive response time. The experimental subjects were divided into the traditional group using traditional agile training methods and the experimental group using agile training equipment, and the following conclusions were obtained after 9 weeks of agile training:

1. The experimental results show that the ESP-MESH-based random distributed agile training instrument designed in this paper has a significant improvement in the agile quality data of experimental subjects trained by traditional methods. It shows that the agile training instrument designed in this paper can improve the sensitivity of college students more efficiently than traditional training methods.
2. According to the experimental data, it can be seen that there is a very significant improvement in the agile training data before and after the training of the traditional group and the experimental group, indicating that whether it is traditional agile training or training with the help of agile training instruments, it can improve the flexibility and coordination of the body by improving the speed and regulation ability of the neuro-muscular system during exercise, thereby improving the sensitivity of college students.
3. By comparing the data of the experimental group after training and the traditional group in the four test schemes, it is found that the former is not as obvious as the other two abilities in changing actions and predicting decision-making ability compared with the latter, because these two abilities are affected by the rest of the physical fitness.

## Data availability statement

The original contributions presented in the study are included in the article/supplementary material, further inquiries can be directed to the corresponding author.

## Ethics statement

Written informed consent was obtained from the individual(s) for the publication of any identifiable images or data included in this article.

## Author contributions

BT, EW, KC, and ST: conceptualization. BT, EW, BZ, LL, and ST: methodology. EW, LX, and ST: software and data curation. EW, ST, LX, KC, BZ, and LL: validation. LL, LX, EW, KC, BZ, and ST: formal analysis. LL, LX, EW, and ST: investigation. BT and LL: resources, supervision, and project administration. BT, EW, LX, and ST: writing-original draft preparation. BT, LL, and ST: writing-review and editing. BT, EW, and ST: visualization. BT: funding acquisition. All authors contributed to the article and approved the submitted version.

## Funding

This work was supported by the Innovation Research and Development Project of the General Administration of Sport of China (22KJCX024), the National Fitness Key Research and Development Project of General Administration of Sport of China (2015B052), the Innovation Fund for Co-Research between Chinese Universities and Industries (2022BL052), Industry-University Cooperation and Education Program of the Ministry of Education (220900487303708) the Major Project of Research on Philosophy and Social Science of Higher Education Institutions in Hubei Province (21ZD054), the Major Project of Hubei Key Laboratory of Intelligent Transportation Technology and Device in Hubei Polytechnic University (2022XZ106), and the Green Technology Leading Program of Hubei University of Technology (CPYF2018009).

## Conflict of interest

The authors declare that the research was conducted in the absence of any commercial or financial relationships that could be construed as a potential conflict of interest.

## Publisher’s note

All claims expressed in this article are solely those of the authors and do not necessarily represent those of their affiliated organizations, or those of the publisher, the editors and the reviewers. Any product that may be evaluated in this article, or claim that may be made by its manufacturer, is not guaranteed or endorsed by the publisher.
